# Preliminary Test of the Reduction Capacity for the Intestinal Adsorption of Skatole and Indole in Weaning Piglets by Pure and Coated Charcoal

**DOI:** 10.3390/ani11092720

**Published:** 2021-09-17

**Authors:** Franziska Witte, Aleksandar Pajic, Florian Menger, Igor Tomasevic, Dana Carina Schubert, Christian Visscher, Nino Terjung

**Affiliations:** 1 German Institute of Food Technologies—DIL e.V., 49610 Quakenbrück, Germany; f.witte@dil-ev.de (F.W.); a.pajic@dil-ev.de (A.P.); 2 Institute of Nutritional and Food Sciences, University of Bonn, 53115 Bonn, Germany; fmenger@uni-bonn.de; 3 Faculty of Agriculture, University of Belgrade, 11080 Belgrade, Serbia; tbigor@agrif.bg.ac.rs; 4 Institute for Animal Nutrition, University of Veterinary Medicine Hanover, 30173 Hanover, Germany; dana.carina.schubert@tiho-hannover.de (D.C.S.); christian.visscher@tiho-hannover.de (C.V.)

**Keywords:** boar taint, charcoal, fat coating, skatole, indole

## Abstract

**Simple Summary:**

To suppress or prevent boar taint, many strategies are available, e.g., surgical or immunocastration or different housing and feeding conditions. In Germany, male piglets may no longer be castrated without anesthesia until the 7th day of life for animal welfare reasons. The aim of this study was to reduce skatole and indole, two of the boar taint-causing substances, by the feeding of charcoal. A fat coating applied on charcoal could delay the unspecific adsorption of charcoal until entry to the large intestine where skatole and indole should be adsorbed since the fat coating is digested during its passage through the small intestine. In total, 18 piglets were divided into three feeding groups and were fed for 19 days with the control feed or with the control feed plus 2% charcoal or plus 4% charcoal coated with a fat layer. The skatole and indole concentrations were analyzed in chyme retrieved from the colon and caecum as these substances need to be adsorbed by charcoal in the intestine to prevent resorption into the bloodstream and accumulation in fat. The reduction in skatole and indole contents underlines charcoal’s adsorption capacity. The adsorption capacity was higher when the charcoal was coated. As charcoal reduced skatole and indole, feeding trials with adult boars are needed to observe the status of boar taint substances at the day of slaughter.

**Abstract:**

To reduce the risk of boar taint, intact male piglets are immuno- or surgically castrated. One alternative is reducing skatole by adding skatole reducing or adsorbing substances to the boars’ diet. Charcoal with a high capacity for adsorbing skatole and indole in vitro (tested before, data not shown) was fed to the boars to test the hypothesis that a fat coating prevents the unspecific adsorption of charcoal before entry into the large intestine while increasing skatole adsorption. Twelve male and six female weaning piglets with initial body weights of 7.74 ± 0.75 kg were fed for 18 (or 19) days with either 2% pure (untreated) charcoal or 4% coated (50% charcoal + 50% fat-coating) charcoal or no charcoal. After euthanasia, skatole and indole were quantified in caecum and colon chyme. Skatole and indole contents in caecum chyme were significantly lower (*p* < 0.05) in the group fed with coated charcoal (33 ± 4.2, 7 ± 2.8 µg/g_DM_, respectively) than in the group fed with pure charcoal (51 ± 7.3, 14 ± 3.0 µg/g_DM_) or with no charcoal (73 ± 12.6, 15 ± 1.7 µg/g_DM_). Similar effects were obvious for colon chyme. The results indicate that a fat coating of charcoal might prevent unspecific adsorption in the small intestine and might consequently lead to a higher adsorption capacity for skatole and indole in the large intestine, as skatole and indole concentrations in the chyme of caecum and colon were approximately 50% lower in the piglets who received coated charcoal.

## 1. Introduction

Entire male pigs offer the advantages of a better feed conversion rate and a higher lean meat content compared to castrates [1]. However, raising entire males is accompanied by the risk of the development of boar taint [2] which is characterized by a fecal or urine-like odour and is caused either by 3-methyl-indole (skatole) or 5α-androstenone (androstenone) alone or by a combination of both [3]. The contribution of indole to boar taint is not clear, as interference between the high indole and skatole contents in pork back fat can occur [4]. In contrast to androstenone, which is formed in the testis and occurs exclusively in boars, skatole and indole are formed by the microbial degradation of L-tryptophan in the large intestine of various monogastric species [1,5]. Nevertheless, especially high skatole concentrations are found in the tissue of boars as androstenone inhibits the hepatic metabolism of skatole [6]. The tryptophan can originate either from apoptotic intestinal epithelial cells or from the feed, particularly when protein sources are used with a low precaecal digestibility [3,7]. Skatole and indole are either excreted in the faeces or resorbed through the intestinal mucosa [8]. If the quantity of the molecules absorbed exceeds the metabolic capacity of the liver, they accumulate in fatty tissue, and to lesser extent in lean meat, or in the liver and kidneys [5]. A positive correlation between skatole concentrations in the adipose tissue and the concentration in the large intestine and blood has been observed [9,10].

The degradation of tryptophan to skatole is induced by the absence of energy in the hindgut and thus it can be suppressed by the diet in combination with rearing and housing regulations [3]. Claus et al. [11] and Zamaratskaia et al. [12] showed a reduction in skatole by feeding raw potato starch to castrates and boars, respectively, whereas indole-producing microbiota remained unaffected [11]. Furthermore, due to inulin from chicory roots [13] or to non-starch polysaccharides [14] from sugar beet [15], skatole concentrations were reduced in boars. On the contrary, Van Oeckel et al. [16] found no reduction in the skatole or in the indole concentrations due to sugar beet pulp, or for wheat bran or soybean hulls. Other feeding approaches with different strategies to reduce boar taint were reviewed by Sander et al. [17]. In an in vivo experiment by Jen and Squires [18], 5% activated carbon as well as 5% tween-60 in the finishing diet of boars reduced androstenone concentrations in plasma and fat.

Charcoal is a heterogeneous substance produced from plant-based materials by pyrolysis between 350 and 1000 °C at low oxygen levels and it is permitted as a feed additive in the EU (No. 68/2013, 7.13.1) [19]. An advantage of using charcoal is the ability to perform ‘enteral dialysis’, but due to the non-specific adsorption of charcoal it can also bind vitamins and nutrients and thus unintentionally channel them out of the animal body [20]. However, the speed of adsorption depends on the pore size [20]. For example, Choi et al. [21] reduced the fecal excretion of Gram-negative coliform bacteria and Gram-negative Salmonella in pigs by a factor of 20 and 1100, respectively, using 0.3% bamboo charcoal and therefore showed the same magnitude of bacterial suppression as the use of antibiotics. In previous investigations, the levels of skatole and indole in the lean meat, fat, liver and chyme of mixed breed piglets were not significantly reduced by adding charcoal to the feed [22]. Nevertheless, no negative effects of charcoal on performance parameters were found either [23].

As charcoal has a non-specific binding capacity, it should be considered that during the passage through the small intestine, the pores of the charcoal might already become loaded with nutrients from the chyme. In order to prevent an unspecific adsorption, a fat coating (melting point > 45 °C) is applied to the charcoal. The fat is expected to be digested by endogenous lipases during its passage through the small intestine so that the charcoal would be free of fat and its pores unloaded when entering the hindgut. Consequently, the aim of the present study was to test how efficiently charcoal adsorbs skatole and indole and if fat-coated charcoal is more efficient than pure charcoal in reducing skatole and indole concentrations in the chyme of weaning piglets. Other feed additives have shown good results but have negative side effects, such as a reduced feed conversion ratio. Piglets were used since they show, independently of gender, a particularly high skatole building potential after weaning and therefore the effect of the charcoal regarding the concentrations of skatole is easier to detect. Additionally, in contrast to boars, the skatole metabolism of piglets is not influenced by androstenone [9,24].

## 2. Materials and Methods

The animal experiments were carried out in accordance with German regulations and were approved by the Animal Welfare Officer of the University of Veterinary Medicine Hanover, Germany (reference: TIHO-T-2019-17, date of approval: 15 September 2019). These animal experiments required no notification or approval in accordance with the Animal Protection Act (§7, paragraph 2, sentence 3). Interventions before dissection were not carried out. The animals were euthanized in accordance with §4, paragraph 3 of the Animal Protection Act, exclusively to use their organs or tissues for scientific purposes.

### 2.1. Animals and Housing

The study was conducted in eighteen mixed breed piglets (sire line: Pietrain; dam line: German Landrace × Large White) including 12 male and 6 females. Females were used since they do not differ from male weaning piglets in terms of skatole and indole metabolism, as weaning piglets show, independently of their gender, a high skatole building potential [24]. The piglets were 25 ± 0.84 days old at the start of the feeding trial and had initial bodyweights (BW) of 7.74 ± 0.75 kg. The animals were housed in 9 pairs, consisting of either 2 males (3 pairs) or 1 male and 1 female (6 pairs). The 1 × 3 m^2^ sized boxes were equipped with a rubber mat as a lying area, infrared warming lamps and environmental enrichment materials. Water was provided by nipple drinkers and the feed was offered from 1 m long metal troughs.

### 2.2. Diets and Feeding Concept

The diets were formulated in order to meet or exceed the nutritional requirements of three- to seven-week-old piglets [25]. The basic feedstuff was based on barley, wheat and soybean meal (Table 1) and modified by either adding 2% pure fat (CON), 2% pure fat and 2% pure charcoal (PURE) or 4% fat-coated charcoal, which consisted of 50% charcoal and 50% fat (COAT) in order to create the final experimental diets and obtain same amounts of nutrients and fat.

The charcoal concentration was based on pre in vitro tests, where concentrations from 0.5 to 5% were investigated (Data not shown). The charcoal (Carbovet, Pancosma, Rolle, Switzerland) and melted fat (Canoletta 77618, Walter Rau, Hilter am Teutoburger Wald, Germany) were extruded in a ratio of 1:1 and subsequently mixed with the other feed material. The chemical composition of the diets can be found in Table 2.

### 2.3. Experimental Procedure

The animals were divided into three homogenous groups (n = 6) according to gender, body weight and litter affiliation. Each group was assigned to one of the three experimental diets for the following 18 or 19 days, respectively. Feeding duration differed as only 12 piglets could be dissected at day 18. However, one pair per feeding group was dissected at day 19. Feed intake was quantified per pair under ad libitum feeding conditions. The body weight was recorded four times for each animal individually at the beginning of each experimental week and in the morning before the dissection.

### 2.4. Dissection

After 18 days, 12 animals and after 19 days, 6 animals, respectively, were dissected. After euthanasia, the chyme of the ascending colon was taken from the flexura centralis coli and from the caecum. The chyme was stored at −18 °C until further analysis.

### 2.5. Sample Preparation and Analyses

The preparation of the samples was conducted by an established method adapted from Gibis et al. [26]. To extract skatole and indole, 1 g chyme (+100 µL 2-Methylindole not used for quantification but as an internal standard for auto sampler and preparation) was vortexed with 20 mL MilliQ water (Merck Millipore, Darmstadt, Germany), centrifuged (9000× *g*, 5 min) and filtered (3 hw, 65 g/m^2^) to gain a 10 mL extract. This extract was then mixed with a 10 mL 0.15 mol/L TRIS-buffer (9.09 g TRIS (Merck, Darmstadt, Germany) + 4.38 g sodium chloride (Sigma-Aldrich, St. Louis, MO, USA), dissolved in MilliQ, adjusted to a pH-value of 8.3, using 2 M hydrochloric acid (Sigma-Aldrich, St. Louis, MO, USA) and filled up to 500 mL with MilliQ, then purified using Oasis HLB cartridges (Waters, Milford, MA, USA), washed with 6 mL MilliQ and eluted with 5 mL methanol (≤100%, Merck KGaA, Darmstadt, Germany). To prevent the injection solvent from having too much eluting power during the measurement, the eluate was mixed with 5 mL MilliQ, transferred in a vial and analyzed by UPLC-MS/MS (ACQUITY-UPLC-System, Waters, MA, USA/API4000, AB Sciex, Framingham, MA, USA). Quantification was completed via an external calibration.

Since skatole and indole quantification were based on the original substance, the chyme was dried at 103 °C for approximately 4 hours in a drying cabinet (Typ B 50, Memmert, Schwabach, Germany) until the weight was constant. Dry matter (DM) was calculated by weight loss and skatole and indole contents were calculated based on the dry matter. For the six replicates (piglets) per feeding group, the chyme was prepared and analyzed in triplicate (biological replicate) (n = 3/chyme).

The diets were analyzed by standard procedures in accordance with the official methods of VDLUFA (Verband deutscher landwirtschaftlicher Untersuchungs- und Forschungsanstalten) [27]. The DM was quantified as described for the chyme. The ash was determined by means of incineration in the muffle furnace at 600 °C after 6 h. The total nitrogen content obtained from the Dumas combustion method (Vario Max ®, Elementar, Hanau, Germany) was multiplied by a factor of 6.25 to receive the crude protein (CP) content. The analysis of the ether extract (EE) was performed after acid hydrolysis in the soxhlet apparatus. To analyse the crude fiber (CF), the samples were washed in dilute acids and alkalis (Fibertec 2010 Hot Extraktor®, Foss, Hilleroed, Denmark). The minerals were quantified by atomic adsorption spectrometry (Unicam Solaar 116, Thermo, Dreieich, Germany).

### 2.6. Skatole and Indole Adsorption In Vitro

For the validation of the charcoal coating in vitro, 2% w/w pure charcoal (PURE), 4% w/w coated charcoal (COAT) or 2% w/w fat (FAT), which was used for the coating, were incubated for 20 hours at 37 °C in 1) colon chyme, 2) caecum chyme and 3) water containing 20 µg/mL indole (I3408-25G, Sigma Aldrich, St. Louis, MO, USA) and 5 µg/mL skatole (M51458-5G, Sigma Aldrich, St. Louis, MO, USA) in triplicate. Skatole and indole adsorption was analyzed as described in 2.5. Colon and caecum chyme were kept as they were and stored at −18 °C until analysis. The conditions of the chyme, such as water content and pH, were not adjusted. The reduction in skatole and indole was calculated from the difference of initial and end content in %. In contrast to in vitro studies with charcoal, e.g., Jen and Squires [28], the feed, its transition time and digestion were neglected as our study aimed to prove the feasibility of the charcoal coating and the difference between pure and coated charcoal. Therefore, caecum and colon chyme were used—without any further modification—for skatole and indole adsorption. To make sure that only charcoal adsorbed skatole and indole, pure fat was used to monitor adsorption. The data produced were used for comparison of PURE to COAT to FAT.

### 2.7. Statistical Analysis

Statistical differences between the mean content of skatole and indole in a feeding group as well as the differences between the feeding groups were analyzed using a One-Way ANalysis Of VAriance (ANOVA) and Tukey’s multiple comparison test in SigmaPlot 14.0 (Systat Software Inc., San Jose, CA, USA). Differences were considered statistically significant when *p* < 0.05. Exact *p*-value are given behind the letter.

## 3. Results and Discussions

### 3.1. Reduction in Skatole and Indole In Vitro

The reduction in indole and skatole by the application of PURE or COAT was significantly (*p* < 0.05) higher in comparison to the FAT application (Table 3). The adsorption of all charcoals was most pronounced in water and less in the colon chyme. The reduced adsorption in chyme could be traced back to other substances, e.g. nutrients or bacteria that block the charcoal’s inner surface [29]. Significant differences (*p* < 0.05) between indole and skatole reduction in the PURE and COAT adsorption capacities between the colon and caecum chyme with water (uppercase letters) underline this. The lower reduction in skatole and indole in water by COAT proved the shielding effect of the fat coating on charcoal. Since fat will not melt <37 °C (melting point: >45 °C) and the adsorption was not 0%, the coating might be incomplete. The shielding and no-melting effect were significant (*p* < 0.05) for the skatole reduction in the colon and tendentially for indole and skatole reduction in the caecum chyme. Still, the difference between PURE and COAT in water was much higher than in chyme.

Even if the piglets’ chyme is utilized in the in vitro trial, the circumstances are different to in vivo, especially regarding the residence time of the feed, the temperature and lipase activity in the intestine as well as other environmental factors (stress, ambient temperature, etc.). Thus, variations between in vivo and in vitro can occur [17]. This difference was also obvious in the study by Jen and Squires, whereby they found less skatole reduction by activated carbon in fat and plasma in vivo [18] than in vitro [28].

The in vitro trial proved that indole and skatole reduction in chyme by charcoal is feasible and showed that fat coating withheld charcoal’s adsorption ability to a certain extent and that fat does not adsorb skatole and indole.

### 3.2. Performance Parameters 

No incidences and no animal losses occurred during the trial. The BW of the three groups did not differ to the beginning or at the end of the trial. At the beginning of the feeding trial, the mean body weight was 7.93 ± 1.01 kg of CON, 7.55 ± 0.59 kg of PURE and 7.75 ± 0.68 kg of COAT. After the feeding period, the body weights were 15.7 ± 2.59 kg, 14.52 ± 1.39 kg and 14.42 ± 1.50 kg, respectively. Daily feed intake (CON: 1049 ± 482; PURE: 942 ± 396; COAT: 943 ± 349 g/d), average daily weight gain (CON: 0.42 ± 0.08; PURE: 0.38 ± 0.05; COAT: 0.36 ± 0.05 kg/d) and feed conversion ratio (CON: 1.18 ± 0.11; PURE: 1.19 ± 0.07; COAT: 1.25 ± 0.10 kg/kg) did not differ among the feeding groups.

### 3.3. Reduction in Skatole and Indole In Vivo

Significant differences (*p* < 0.05) were found between the skatole concentrations in the colon and between indole and skatole in the caecum chyme (Table 4). Probably due to the high indole content in the colon chyme of the sixth piglet in the COAT group, this group did not differ to PURE and CON. In the caecum chyme, no high indole levels for this sixth piglet from COAT were found. According to Lanthier et al. [30], variations in skatole content are a ‘dynamic phenomenon’ as skatole is influenced by more factors than feed, stress, sex and age. Skatole contents are individual, especially for piglets after weaning [30]. Thus, Lanthier, Lou et al. [30] concluded that in vivo studies with weaning pigs have limited use in relation to the study of boar taint generally, but as weaning piglets have the highest skatole building potential [24], the feasibility of feed additives for skatole and indole reduction can be investigated. In line with our results, Claus, Lösel et al. [11] and Zamaratskaia, Babol et al. [12] found that skatole was reduced by feeding raw potato starch, whereas indole remained at the same level. In line with our findings, Øverland, Kjos et al. [2] found less skatole in the caecum than in the colon chyme but similar indole content in the colon and caecum chyme. 

Norwegian studies [2,31] have shown lower skatole contents in caecum and colon chyme due to the addition of about 6% of inulin for at least 7 to 30 days or 20% of raw potato starch for 16 days to the boars’ diet. Lanthier, Lou et al. [24] investigated the skatole content in the plasma of prepubescent pigs at different times postweaning and found that the addition of 10% of inulin in the piglets’ diet suppressed the actual increase in the skatole in the plasma. Moreover, due to the feeding of 160 g/kg sugar-beet pulp, the skatole and indole contents in the faeces as well as the blood were significantly lower compared to the 87 g/kg intake [14].

Generally, PURE and COAT reduced the skatole and indole concentrations in the caecum and colon chyme by adsorption. However, the skatole and indole contents in the caecum chyme of COAT were significantly (*p* < 0.05) reduced in comparison to PURE (indole) and CON (skatole), supposedly due to the withholding of charcoal’s adsorption capacity until fat degradation. This statement is supported by similar tendencies in colon chyme, proving our hypothesis.

The use of sorbent materials, such as clinoptilolite [32], zeolite [33] and charcoal [18] to adsorb skatole in finishing boars intestinally have shown contradictory results: 0.5% zeolite (≙0.45% clinoptilolite) did reduce skatole significantly in adipose tissue [33], whereas 1% clinoptilolite [32] and 5% activated carbon [18] have not been proven to reduce skatole. Jen and Squires [18] assumed that they found no effect as the skatole concentrations were low in all feeding groups. However, the authors found reduced androstenone concentrations in plasma and back fat [18]. Considering that feeding with 5% activated carbon is possible [18], feeding trials with higher concentrations of charcoal would be interesting. Although feeding charcoal is considered safer [20] and cheaper compared to other feed additives that aim to reduce boar taint, economic factors such as price per kilo, addition to feed, daily growth and animal welfare need to be observed—always in relation to the application time. Yet, based on our findings, charcoal represents an option that is available worldwide that can reduce skatole and indole in the intestine with the aim of reducing boar taint without affecting daily feed intake, average daily weight gain and the feed conversion ratio as shown in this study. Moreover, feeding charcoal can increase feed intake, weight gain, feed efficiency, improve the meat quality, strengthen the immune system and as no negative side effects are known, can reduce veterinary costs. In addition, excreted faeces with charcoal can be a valuable organic fertilizer resulting in lower greenhouse gas emissions [20].

## 4. Conclusions

Skatole and indole contents in caecum chyme were significantly lower in the group fed with coated charcoal than in the groups that were fed with pure charcoal or control feed and similar tendencies are obvious for colon chyme. Thus, charcoal’s adsorption capacity might be shielded until fat degradation in the intestine. Additional investigations with adult boars are needed to confirm the reduction in the overall boar taint and to investigate the long-term effects of charcoal on animal welfare as well as on meat quality.

## Figures and Tables

**Table 1:** Composition (%) of the of the basis feed for the experimental diets.

Item	Percentage
Barley	35.0
Wheat	16.0
Soybean meal *	15.0
Maize	15.0
Waffle meal	4.0
Wheat bran	4.0
Beet pulp	1.0
Sunflower extraction meal	1.0
Fish protein concentrate	1.0
Premix **	8.0

* Soybean meal made from genetically modified soybeans. ** Contains fats and oils; additives (per kg feed); nutritional additives: vitamin A (10,000 IU), vitamin D/vitamin D3 (1675 IU), vitamin E (80 mg), iron from iron-(II)-sulfate monohydrate (104 mg), copper from copper-(II)-sulfate pentahydrate (8 mg), copper from copper chelate of the hydroxyl analogue of methionine (4 mg), manganese from manganese-(II)-sulfate (46 mg), manganese from manganese chelate of the hydroxyl analogue of methionine (8 mg), zinc from zinc sulfate monohydrate (67 mg), zinc from zinc chelate of the hydroxyl analogue of methionine (17 mg), iodine from calcium iodate anhydrous (1.7 mg), selenium from sodium selenite (0.21 mg), selenium methionine from *Saccheromyces cerevisiae* (0.08 mg).

**Table 2:** Chemical composition of the three experimental diets CON (control diet), PURE (diet containing pure charcoal) and COAT (diet containing coated charcoal).

		CON	PURE	COAT
Organic matter	g/kg DM	947	947	946
Crude protein		192	186	189
Ether extract		50.6	61.2	51.8
Crude fibre		52.4	55.7	53.3
NfE ^1^		652	644	652
Calcium		7.17	7.55	7.75
Phosphorus		5.43	5.34	5.53
Potassium		7.22	7.10	7.09
Magnesium		2.16	2.12	2.16
Copper	mg/kg DM	51.5	49.5	51.2
Zinc		153	164	152
Iron		409	455	406

^1^ Nitrogen-free extract (NfE) = dry matter − (ash + crude protein + ether extract + crude fibre).

**Table 3:** Indole and skatole reduction (%) in vitro (mean ± standard error of the mean) by pure charcoal (2% w/w) (PURE), coated charcoal (4% w/w) (COAT) and fat (2% w/w) (n = 3).

	Indole Reduction (%)						Skatole Reduction (%)					
	PURE		COAT		FAT		PURE		COAT		FAT	
Colon chyme	18.80 ± 4.22	^a 0.005^	14.53 ± 0.73	^a 0.019^	1.00 ± 0.00	^b^	17.57 ± 1.82	^a 0.007 (to b)^	9.17 ± 1.12	^b 0.008 (to c)^	1.00 ± 0.00	^c < 0.001 (to a)^
Caecum chyme	24.40 ± 0.81	^a < 0.001^	17.53 ± 3.20	^a 0.003^	1.60 ± 0.60	^b^	22.10 ± 0.32	^a < 0.001^	14.20 ± 3.31	^a 0.007^	1.00 ± 0.00	^b^
Water	90.03 ± 0.20	^a <0.001^	19.80 ± 1.08	^b < 0.001^	1.00 ± 0.00	^c < 0.001^	99.73 ± 0.09	^a < 0.001^	33.43 ± 2.97	^b < 0.001^	1.00 ± 0.00	^c < 0.001^

Different lowercase letters in a row indicate significant differences between either indole or skatole reduction among PURE, COAT and FAT in colon or caecum chyme or water. Significant differences were determined using One-Way ANOVA and Tukey test (*p* < 0.05). Exact *p*-values are given behind the letter and refer to the deviating letter in a row.

**Table 4:** Indole and skatole (µg/g DM (Dry matter)) contents in vivo (mean ± standard error of the mean) in colon and caecum chyme from piglets of CON (control diet), PURE (diet containing pure charcoal) and COAT (diet containing coated charcoal) (n = 3).

	Colon Chyme						Caecum Chyme					
	Indole (µg/g DM)			Skatole (µg/g DM)			Indole (µg/g DM)			Skatole (µg/g DM)		
	CON	PURE	COAT	CON	PURE	COAT	CON	PURE	COAT	CON	PURE	COAT
	8.11 ± 0.69 ^a^	12.28 ± 1.28 ^a^	1.32 ± 0.00 ^a^	62.39 ± 3.83 ^a c^	21.51 ± 1.84 ^a^	1.32 ± 0.00 ^a^	41.30 ± 1.80 ^a^	15.65 ± 3.39 ^a^	27.86 ± 1.88 ^a^	13.83 ± 0.08 ^a^	2.04 ± 0.00 ^a^	2.91 ± 0.00 ^a^
	12.18 ± 0.95 ^a^	1.40 ± 0.00 ^b^	3.08 ± 0.41 ^a^	169.01 ± 11.61 ^b^	84.11 ± 9.40 ^b^	51.68 ± 4.53 ^b^	9.26 ± 1.19 ^b^	2.48 ± 0.00 ^b^	2.68 ± 0.49 ^b^	26.93 ± 6.27 ^a^	3.67 ± 0.60 ^a b^	2.30 ± 0.11 ^a^
	8.83 ± 0.25 ^a^	5.14 ± 1.02 ^b^	1.66 ± 0.00 ^a^	38.01 ± 1.45 ^a^	35.17 ± 2.66 ^a^	38.43 ± 0.49 ^c^	5.90 ± 1.11 ^b^	12.72 ± 0.87 ^a b^	2.38 ± 0.00 ^b^	2.27 ± 0.00 ^a^	2.04 ± 0.00 ^b^	2.63 ± 0.18 ^a^
	23.46 ± 3.32 ^b^	2.30 ± 0.58 ^b^	1.59 ± 0.00 ^a^	73.31 ± 5.08 ^c e^	85.28 ± 6.43 ^b^	46.72 ± 2.28 ^bc^	8.22 ± 0.50 ^b^	38.20 ± 1.90 ^c^	2.11 ± 0.00 ^b^	81.16 ± 5.84 ^b^	46.43 ± 3.47 ^c^	13.90 ± 2.40 ^b^
	24.12 ± 1.83 ^b^	27.57 ± 2.06 ^c^	1.55 ± 0.00 ^a^	4.18 ± 0.43 ^d^	69.02 ± 5.10 ^b^	20.57 ± 1.59 ^d^	23.93 ± 4.65 ^a b^	45.82 ± 4.92 ^c^	2.36 ± 0.14 ^b^	2.44 ± 0.00 ^a^	2.94 ± 0.00 ^a b^	2.22 ± 0.00 ^a^
	14.38 ± 1.41 ^a^	33.02 ± 1.98 ^c^	32.19 ± 3.31 ^b^	95.83 ± 6.82 ^e^	13.33 ± 0.80 ^a^	37.29 ± 2.11 ^c^	48.23 ± 5.30 ^a b^	11.17 ± 1.16 ^a b^	10.35 ± 1.27 ^c^	18.38 ± 2.34 ^a^	2.83 ± 0.37 ^a b^	2.59 ± 0.00 ^a^
Mean	15.18 ± 1.67 ^A^	13.62 ± 3.04 ^A^	6.90 ± 2.79 ^A^	73.79 ± 12.62 ^A^	51.40 ± 7.32 ^AB^	32.67 ± 4.21 ^B^	18.25 ± 3.62 ^AB^	21.01 ± 3.87 ^A^	7.96 ± 2.30 ^B^	22.48 ± 6.82 ^A^	9.99 ± 3.99 ^AB^	4.42 ± 1.08 ^B^

Different superscript lowercase letters in a column indicate significant differences between either indole or skatole content in either colon or caecum chyme of animals of one feeding group. Different upper-case letters in the row ‘mean’ for either indole or skatole content of either colon or caecum chyme indicate significant differences between feeding groups. Significant differences were determined using One-Way ANOVA and Tukey test (*p* < 0.05).

## Data Availability

The data presented in this study are available in this manuscript.
